# Supplementary figures and images for: Intestinal Serum amyloid A suppresses systemic neutrophil activation and bactericidal activity in response to microbiota colonization
Source: PLoS Pathog. 2019 Mar 7;15(3):e1007381. doi: 10.1371/journal.ppat.1007381 (PMC6405052; doi:10.1371/journal.ppat.1007381)

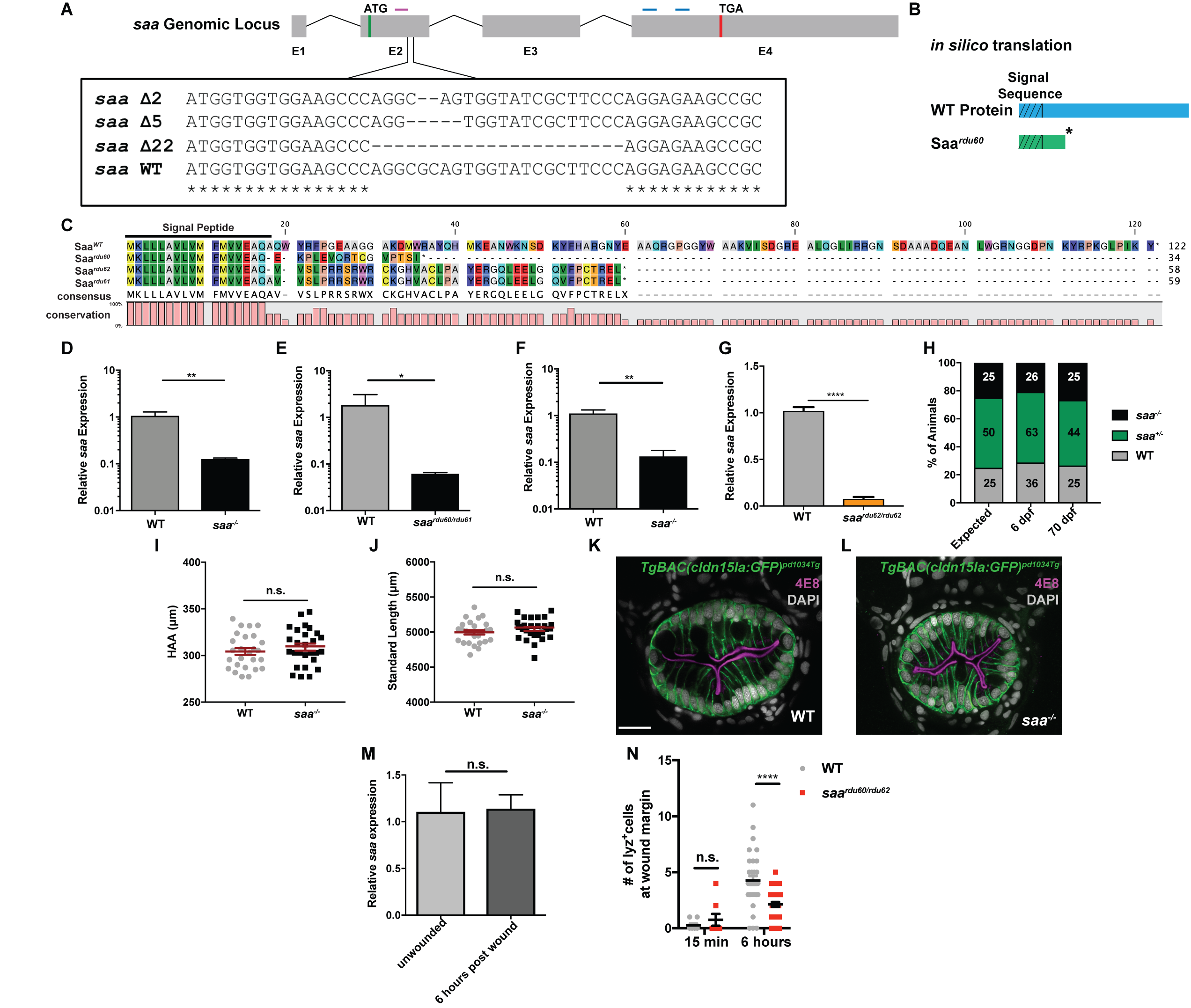

Supplement: S1 Fig — (A) Sequence of CRISPR/Cas9 induced deletions in the coding region of zebrafish saa exon 2. (B-C) In silico translation of mutant allele rdu60 revealed a frame shift mutation in exon 2 with a predicted early stop codon, indicated by the asterisk. (D) qRT-PCR of 6 dpf whole larvae demonstrated negligible saa mRNA expression in saa homozygous mutant zebrafish (saardu60/rdu60 or saa-/-). (E) qRT-PCR of 6 dpf whole larvae from trans-heterozygous saardu60/rdu61 in-crosses revealed reduced saa mRNA levels relative to WT controls. (F) qRT-PCR of dissected digestive tracts from 6 dpf WT and saa-/- larvae demonstrated significantly reduced saa mRNA levels. (G) qRT-PCR of 6 dpf whole larvae from homozygous saardu62/rdu62 in-crosses demonstrated reduced saa mRNA levels relative to WT controls. (qRT-PCR shown in panels D-G included 4–8 replicates / genotype, n ≥ 20 larvae / replicate). (H) Representative genotype distributions exhibited no deviation from expected Mendelian outcomes indicating no difference in viability of saa-/- animals (p = 0.8257) (from two independent experiments, actual number of animals from each genotype overlaid on bars). (I-J) Morphometric analysis illustrated loss of saa does not impact growth [standard length (SL), height at anterior of anal fin (HAA)] in 6 dpf larvae (n ≥ 26 larvae). (K-L) Confocal micrographs of transverse sections from transgenic WT and saa-/- 6 dpf larvae positive for TgBAC(cldn15la:EGFP)pd1034Tg (which labels IEC basolateral membranes with a Cldn15la-GFP fusion protein), and immunofluorescence labeling with the brush-border antibody 4E8 demonstrated intestinal architecture is qualitatively normal in mutant animals (scale bar = 50 μm). (M) qRT-PCR of 6 dpf whole larvae following a tail amputation showed no significant induction of saa. (N) lyz:GFP+ neutrophil recruitment to caudal fin wound 6 hours following amputation in 6 dpf zebrafish larvae revealed decreased neutrophil recruitment in saa compound mutants (saardu60/rdu62) vers [file ppat.1007381.s001.tif]

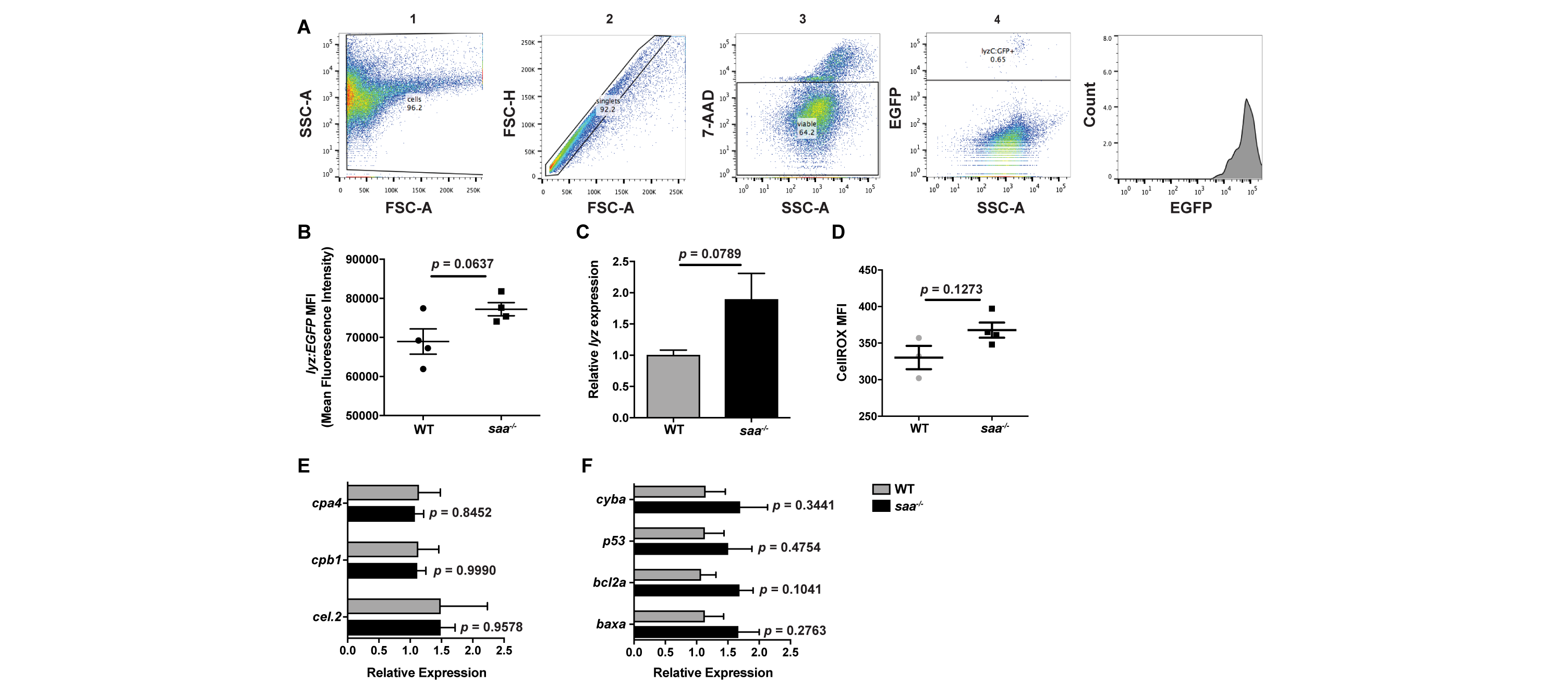

Supplement: S2 Fig — (A) Gating strategy for isolation of lyz:EGFP+ neutrophils from 6 dpf zebrafish larvae. (B) The mean fluorescence intensity (MFI) of the lyz:EGFP+ neutrophil population was not significantly different between WT and saa mutant larvae. (C) qRT-PCR revealed no significant difference in lysozyme C (lyz) transcript levels in sorted lyz+ neutrophils from WT and saa mutant larvae. (D) Quantification of intracellular ROS levels as indicated by CellROX staining measured by flow cytometry in lyz:EGFP+ neutrophils from WT and saa mutant larvae showed no significant difference. (E-F) qRT-PCR analysis of sorted neutrophils revealed no differential expression of genes associated with pro-myelocyte progenitors (cpa4, cpb1, cel.2) or apoptotic markers (cyba, p53, bcl2a, baxa) between WT or saa mutants. (For panels B—F: n ≥ 4 replicates / genotype, n = 60–90 larvae / genotype). In panels B-F data was analyzed by t-test. Data are presented as mean ± SEM. * p < 0.05, ** p < 0.01, *** p < 0.001, **** p < 0.0001. (TIF) [file ppat.1007381.s002.tif]

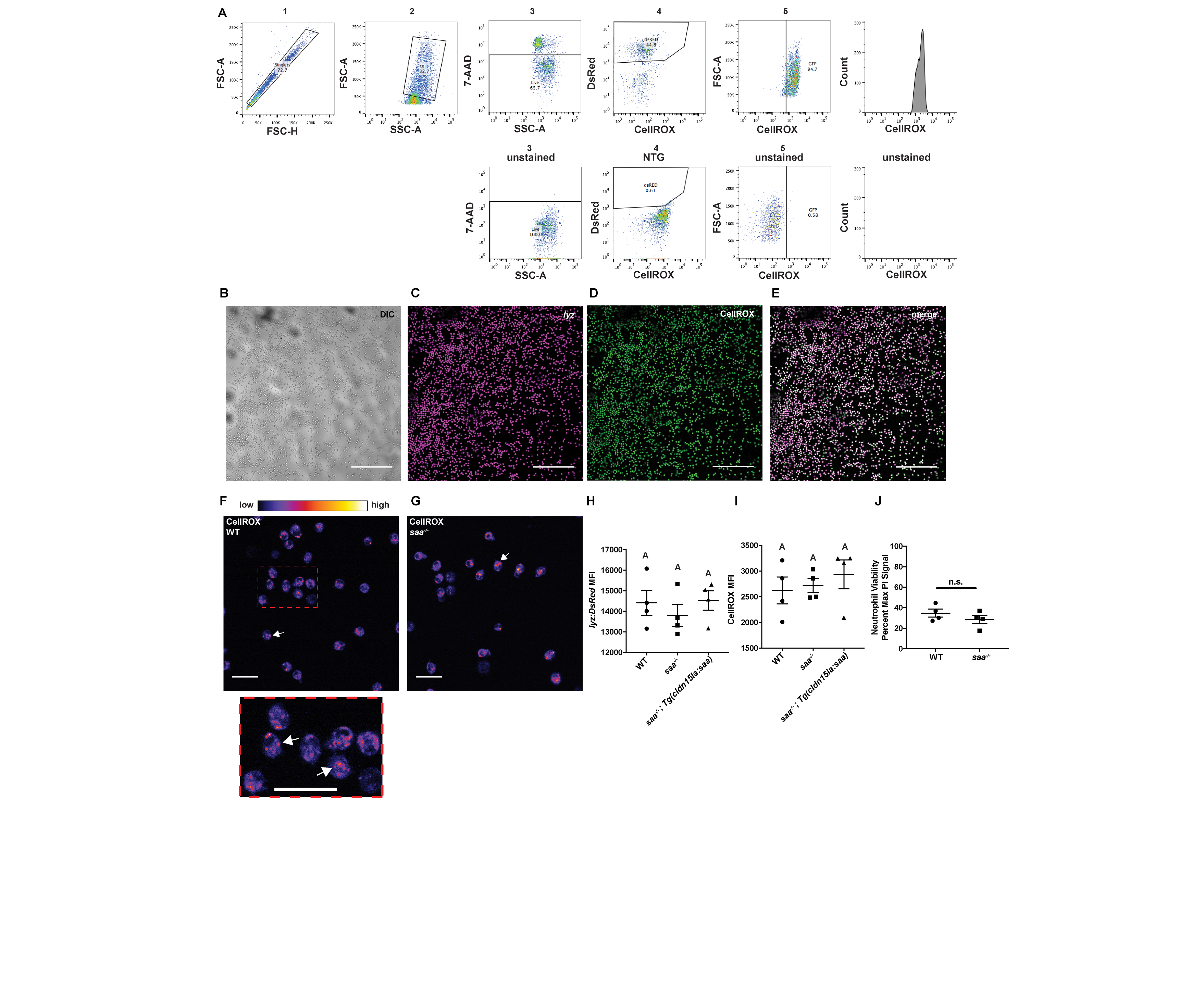

Supplement: S3 Fig — (A) Gating strategy for isolation of CellROX labeled lyz:DsRed+ neutrophils from adult zebrafish kidneys. (B-E) Low magnification (10x) confocal images of lyz:EGFP+ neutrophils labeled with CellROX ex vivo (scale bar = 200 μm). (F-G) Imaging of CellROX labeled lyz:EGFP+ neutrophils isolated from WT and saa mutant zebrafish revealed cytoplasmic punctae (indicated by white arrows). Red dashed box indicates region enlarged to show cytoplasmic CellROX punctae (Scale bar = 20 μm). (H) The mean fluorescence intensity (MFI) of the lyz:DsRed+ population was unchanged between WT, saa-/-, or saa-/-;Tg(cldn15la:saa) neutrophils (4 replicates / genotype). (I) Quantification of intracellular ROS levels as indicated by CellROX staining measured by flow cytometry of lyz:DsRed+ neutrophils illustrated no significant difference in baseline CellROX levels between genotypes (4 replicates / genotype). (J) Measurement of lyz:EGFP+ neutrophil viability as assessed by Propidium Iodide (PI) staining demonstrated no significant differences between genotypes after 4 hours of co-culture with E. coli (relative to maximum PI signal from lysed neutrophils) (4 replicates / genotype). In panels H-I, data were analyzed by one-way ANOVA with Tukey’s multiple comparisons test. In panel J, data were analyzed by t-test. Data are presented as mean ± SEM. * p < 0.05, ** p < 0.01, *** p < 0.001, **** p < 0.0001. (TIF) [file ppat.1007381.s003.tif]

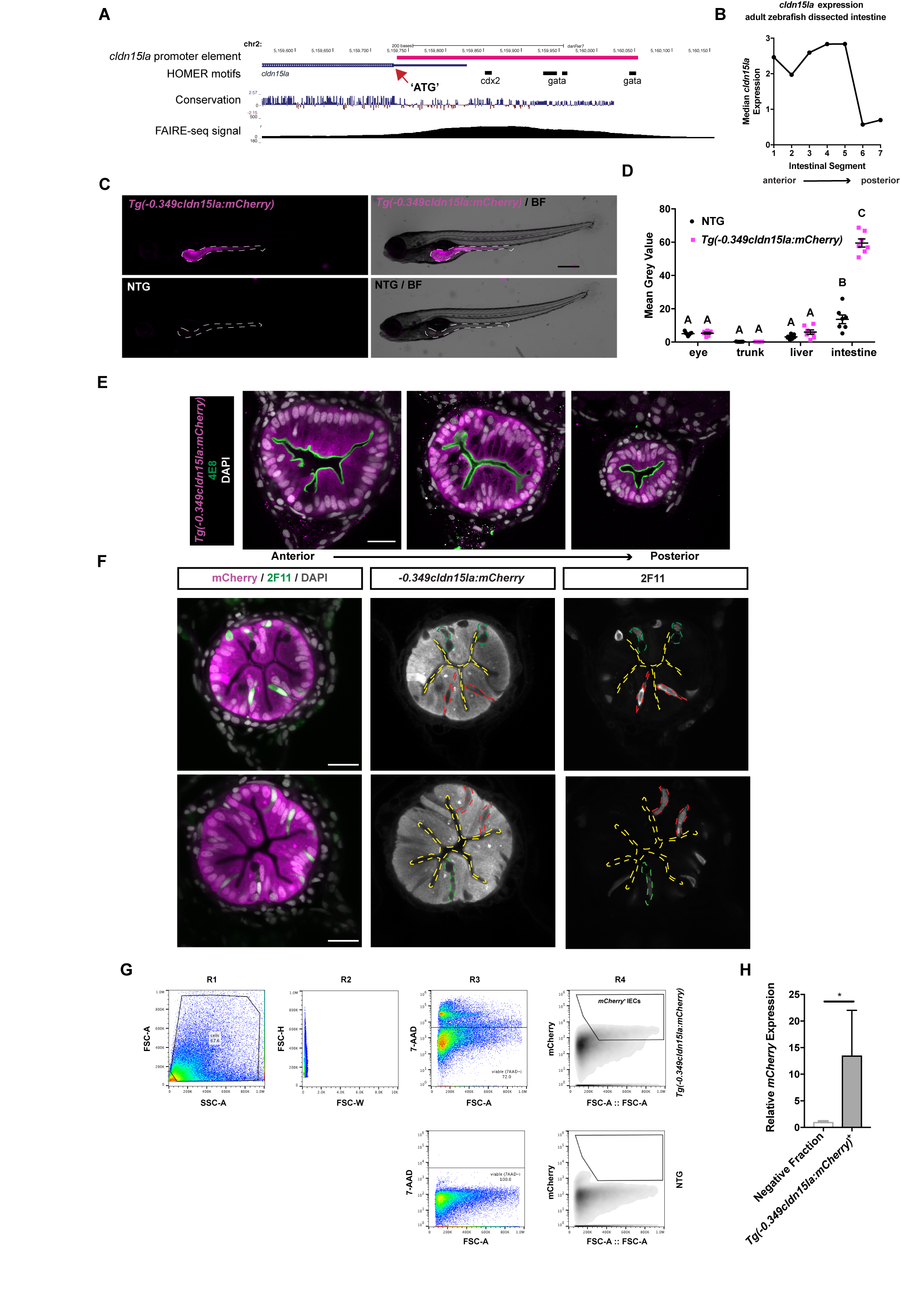

Supplement: S4 Fig — (A) UCSC genome browser view of the zebrafish cldn15la gene locus with the translational start indicated by the red arrow. Pink bar represents the cloned 349 bp promoter region upstream of the cldn15la gene used to drive intestine-specific transgene expression. Tracks for vertebrate conservation, FAIRE-seq and motifs for transcription factors important of IEC gene expression programs (identified by HOMER) are shown below the locus [60]. (B) Expression pattern of endogenous cldn15la along the length of the intestine in adult zebrafish, as measured by microarray in Wang et al., 2010 [59]. (C) Representative stereoscope images of IEC specific cytosolic mCherry expression in 5 dpf Tg(-0.349cldn15la:mCherry)rdu65 larvae compared to non-transgenic (NTG) controls (scale bar = 500 μm). White dashed line indicates the intestine. (D) Quantification of mCherry fluorescence in the indicated tissues of 6 dpf Tg(-0.349cldn15la:mCherry) and non-transgenic control larvae demonstrated intestine-restricted mCherry reporter activity (n = 7 larvae / genotype). (E) Representative confocal micrographs of immunostained transverse sections of Tg(-0.349cldn15la:mCherry) 6 dpf larvae along the anterior-posterior axis labeled with the absorptive cell brush border-specific antibody 4E8 illustrated transgene expression in absorptive enterocytes (scale bar = 20 μm). (F) Representative immunofluorescence images of transverse sections from Tg(-0.349cldn15la:mCherry) 6 dpf larvae stained with secretory cell-specific antibody 2F11 demonstrated weak expression in secretory cells, including enteroendocrine cells (outlined by red dashed lines) and goblet cells (outlined by green dashed lines) (scale bar = 20 μm). (G) Gating strategy for isolation of -0.349cldn15la:mCherry+ IECs from larval zebrafish. (H) qPCR for mCherry transcript in sorted Tg(-0.349cldn15la:mCherry) IECs showed enrichment as compared to negative fraction (13,000 –0.349cldn15la:mCherry+ or mCherry negative cells / replicate, 4 replica [file ppat.1007381.s004.tif]

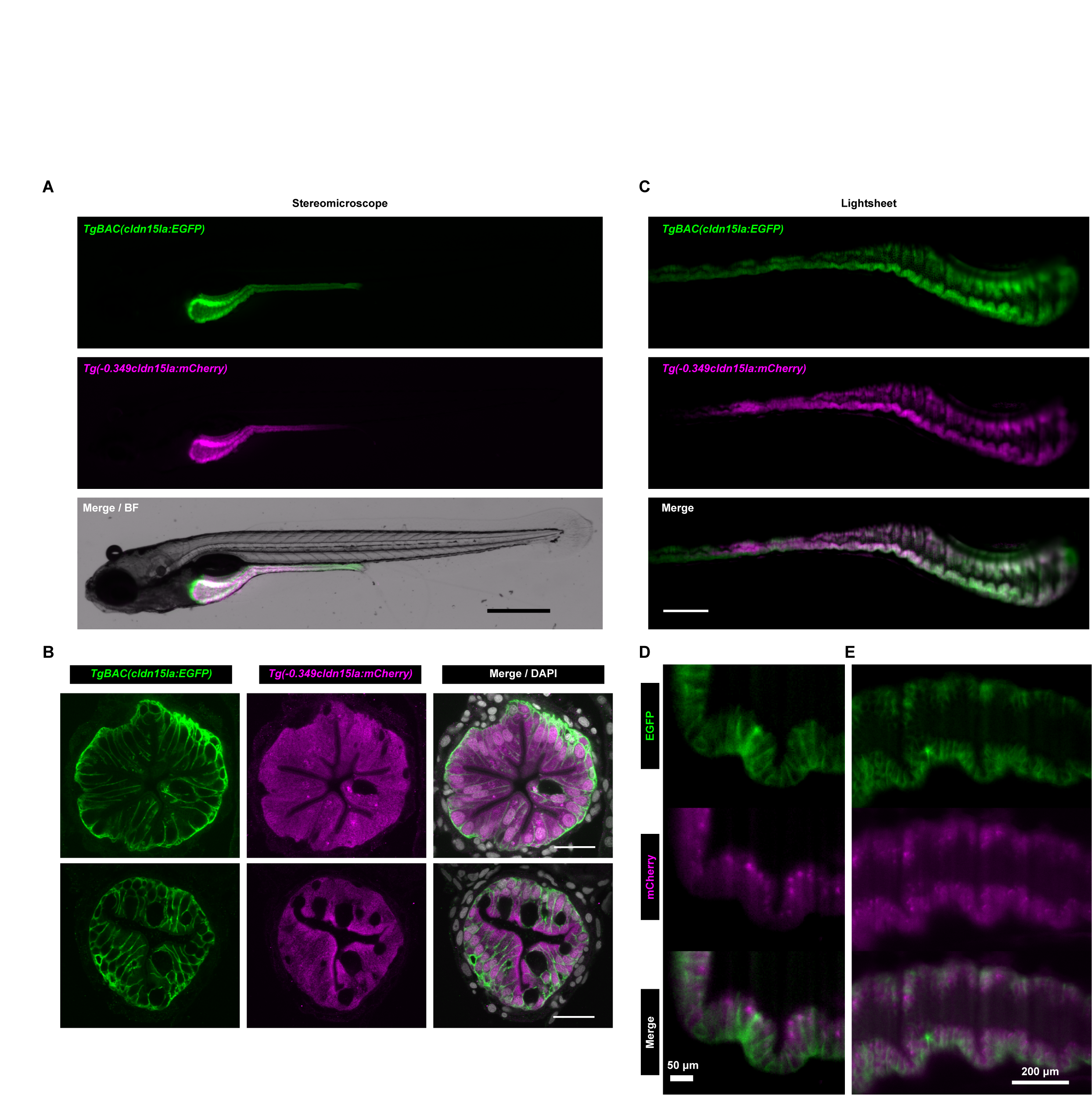

Supplement: S5 Fig — (A) Widefield fluorescence images of IEC-specific cytosolic mCherry expression in 6 dpf double transgenic Tg(-0.349cldn15la:mCherry)rdu65; TgBAC(cldn15la:EGFP)pd1034Tg larvae demonstrated overlap in mCherry and GFP expression domains (scale bar = 500 μm). (B) Representative confocal micrographs of immunolabeled transverse sections from anterior (upper) and posterior (lower) intestinal segments of Tg(-0.349cldn15la:mCherry)rdu65;TgBAC(cldn15la:EGFP)pd1034Tg 6 dpf larvae revealed expression of both mCherry and EGFP in IECs (scale bar = 20 μm). (C) Representative maximum intensity projections from single plane illumination microscopy (SPIM) z-stacks of Tg(-0.349cldn15la:mCherry)rdu65;TgBAC(cldn15la:EGFP) pd1034Tg 6 dpf larvae (scale bar = 500 μm). (D-E) High magnification single slice lightsheet images of the intestinal epithelium in a representative 6 dpf Tg(-0.349cldn15la:mCherry)rdu65;TgBAC(cldn15la:EGFP) pd1034Tg larva. (TIF) [file ppat.1007381.s005.tif]

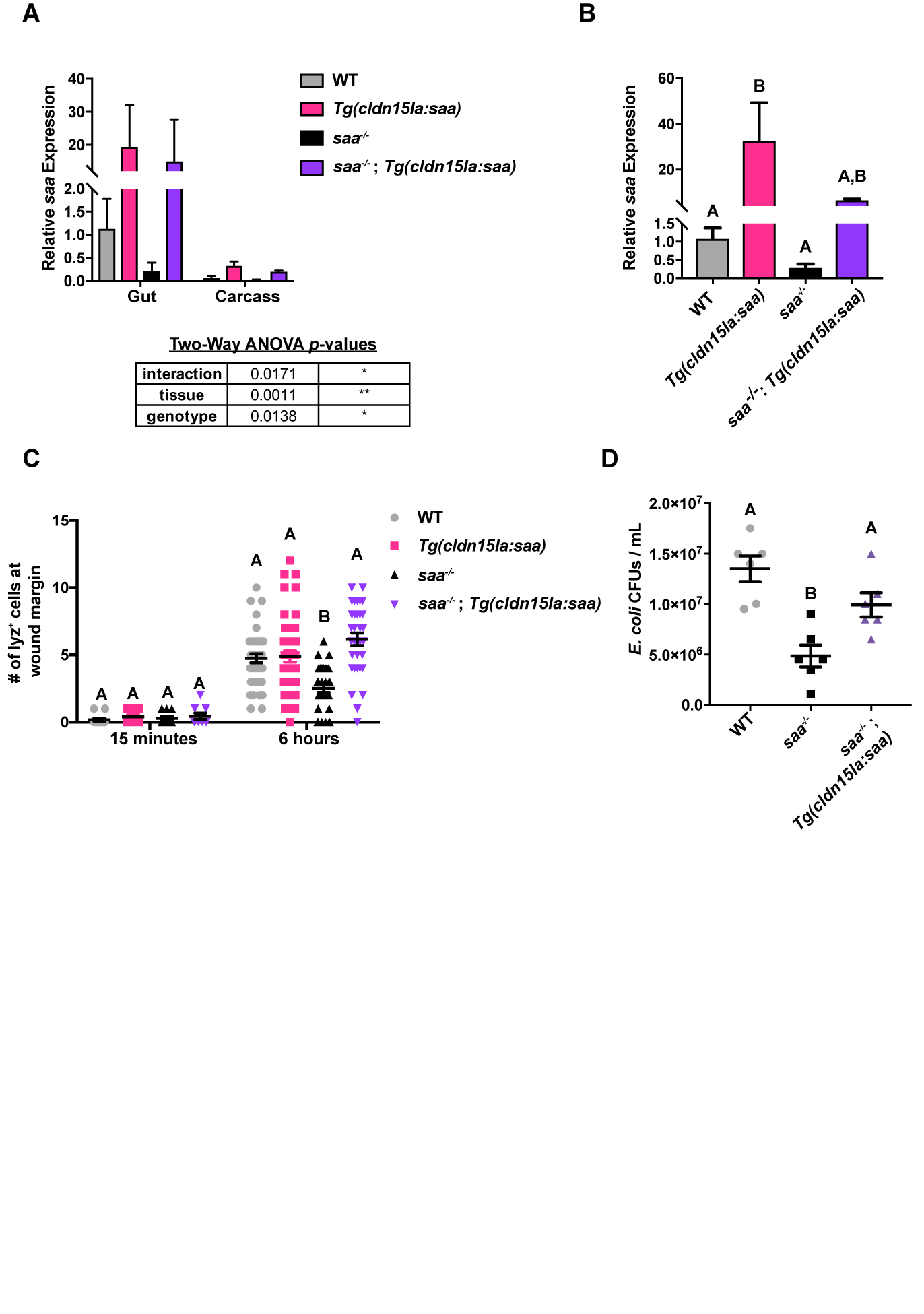

Supplement: S6 Fig — (A) qRT-PCR analysis of dissected digestive tracts and carcasses (remaining tissues following removal of the intestine) from 6 dpf larvae of indicated genotypes revealed significant increase in saa expression in transgenic larvae is restricted to the gut (4 replicates / genotype, 20 larvae / replicate). [73] (B) qRT-PCR of saa from whole 6 dpf larvae of the indicated genotypes (n = 4 replicates / genotype, 15–20 larvae / replicate). (C) Enumeration of lyz:DsRed+ wound-associated neutrophils at 6 hours post amputation revealed intestinally-derived saa does not affect neutrophil recruitment in WT larvae (n ≥ 32 larvae / genotype at 6 hour time point). (D) CFU quantification of bacterial concentration following 4 hour co-culture of lyz:DsRed+ adult zebrafish neutrophils with E. coli (MOI 2) (3–6 replicates / genotype). Data in panel A analyzed by a two-way ANOVA with p values reported in the table. Data in panel B was analyzed with a Kruskal-Wallis test. Data in panels C-D analyzed by one-way ANOVA with Tukey’s multiple comparison’s test. Data are presented as mean ± SEM. * p < 0.05, ** p < 0.01, *** p < 0.001, **** p < 0.0001. (TIF) [file ppat.1007381.s006.tif]

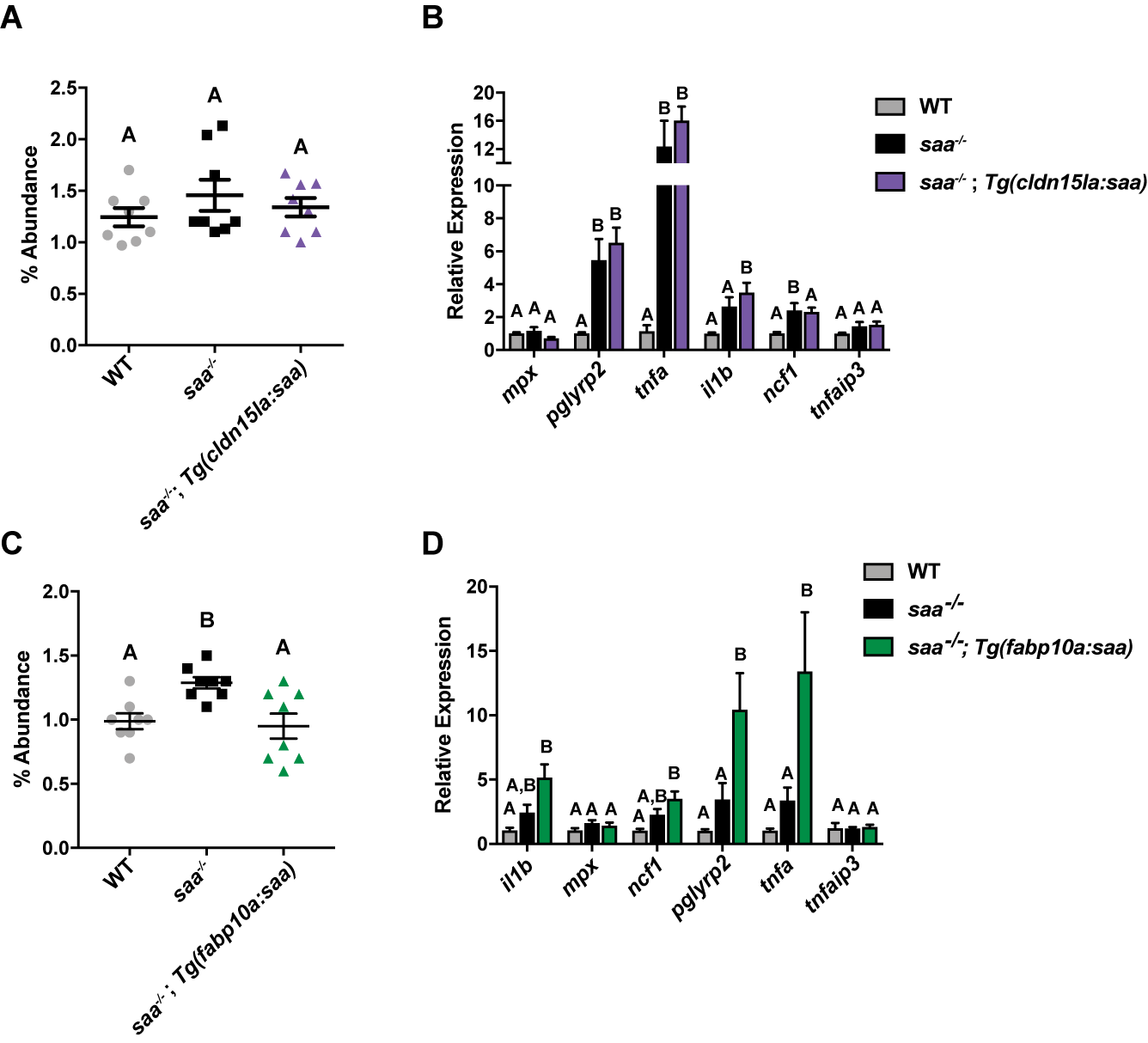

Supplement: S7 Fig — (A) FACS revealed no significant difference in abundance of lyz:DsRed+ neutrophils in 6 dpf saa-/- larvae as compared to saa-/-;Tg(cldn15la:saa) larvae (4 replicates / genotype / experiment, n ≥ 60 larvae / replicate, data pooled from 2 independent experiments). (B) qRT-PCR of pro-inflammatory mRNAs from lyz:DsRed+ neutrophils isolated from both saa-/- and saa-/-;Tg(cldn15la:saa) larvae revealed persistent transcriptional activation as compared to WT (4 replicates / genotype, n ≥ 60 larvae / replicate). (C) FACS demonstrated increased abundance of lyz:DsRed+ neutrophils in 6 dpf saa-/- larvae which is restored to WT levels in saa-/-;Tg(fabp10a:saa) larvae (4 replicates / genotype / experiment, n ≥ 60 larvae / replicate, data pooled from 2 independent experiments). (D) qRT-PCR of pro-inflammatory mRNAs from lyz:DsRed+ neutrophils isolated from both saa-/- and saa-/-;Tg(fabp10a:saa) larvae revealed persistent transcriptional activation as compared to WT (4 replicates / genotype, n ≥ 60 larvae / replicate). Data in panels A-D were analyzed by one-way ANOVA with Tukey’s multiple comparisons test. Data are presented as mean ± SEM. * p < 0.05, ** p < 0.01, *** p < 0.001, **** p < 0.0001. (TIF) [file ppat.1007381.s007.tif]

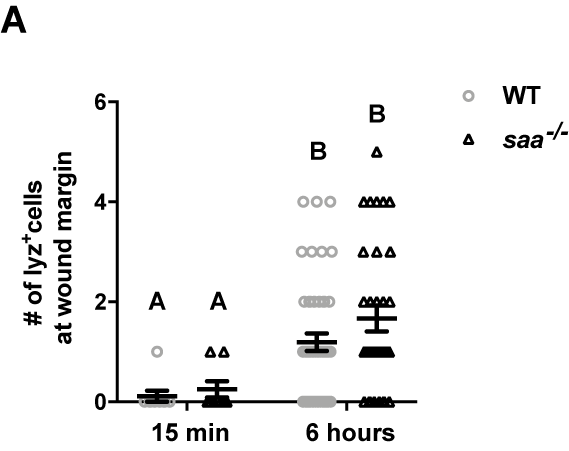

Supplement: S8 Fig — (A) lyz:EGFP+ neutrophil recruitment to caudal fin wound margin 6 hours after amputation in 6 dpf gnotobiotic GF WT and GF saa-/- zebrafish larvae was not significantly different (n ≥ 33 larvae / genotype / condition at the 6 hour timepoint). Data analyzed by a two-way ANOVA. (TIF) [file ppat.1007381.s008.tif]
