# Supplementary material for: Intestinal Serum amyloid A suppresses systemic neutrophil activation and bactericidal activity in response to microbiota colonization
Source: PLoS Pathog. 2019 Mar 7;15(3):e1007381. doi: 10.1371/journal.ppat.1007381 (PMC6405052; doi:10.1371/journal.ppat.1007381)
Supplement: S1 Table — (PDF) [file ppat.1007381.s009.pdf]

S1 Table. Primers used in this study (qRT-PCR, cloning and genotyping).

	**Oligos for cloning guide RNAs**		
P1	SAA gRNA ex2a F	TAGGAAGCGATACCACTGCGCC	
P2	SAA gRNA ex2a R	AAACGGCGCAGTGGTATCGCTT	
	**Primers for Saa mutation screening and genotyping**		
P3	crisprSAA F1	CATGAAGCTTCTTCTTGCTGTGCTGG	
P4	crisprSAA R1	GGAATAACAACTGACCTCCAGCGGCTTCTCC	
P9	saa genotyping R2 intron	CATAACACTATCAAGTTGTCCCAAATTGTG	
	**Primers for cloning danio rerio cldn15la promoter**		
P5	dr cldn15laF	AAGGCCGGCCGCTGTTCACAGTGATTCCCTCACTAATGG	
P6	dr cldn15laR	AAGGCGCGCCCAACCTCAGAAATCCAAAATTCAAGGGAAG	
	**Primers for cloning danio rerio *saa* CDS**		
P7	drSAA pME F:	ccgccccccttcaccATGAAGCTTCTTCTTGCTGTGCTGG	
P8	drSAA pME R:	tcggcgcgcccacccttTCAGTACTTTATGGGCAGGCCTTTAG	

**qRT-PCR Primers**		
Dr_l-plastin_F	TGTCTGTGCCCGACACCAT	Kanther et al., 2014
Dr_l-plastin_R	GGCGGAGGCAGAGTTCAG	Kanther et al., 2014
Dr_gcsfa_F	GCTTTTTGATTGGTGTTGCTATAATG	Statchura et al., 2013
Dr_gcsfa_R	CAACGATCCCCACTAATGTGAA	Statchura et al., 2013
Dr_elfa_F	CTTCTCAGGCTGACTGTGC	Marjoram et al., 2015
Dr_elfa_R	CCGCTAGGATTACCCTCC	Marjoram et al., 2015
Dr_nfkbiaa_F	GCCGGACAGCCCTTAAATTC	Kanther et al., Gastro., 2011
Dr_nfkbiaa_R	TCATAAATACAATCGTCCTCAGACATG	Kanther et al., Gastro., 2011
Dr_lyzC_F	TCGTGTGAAAGCAAGACACTGGGA	Kanther et al., Gastro., 2011
Dr_lyzC_R	ACTCGGTGGGTCTTAAACCTGCTT	Kanther et al., Gastro., 2011
Dr_pglyrp2_F	TGCAGGAGGATTTCACCATTC	Oehlers et al., 2011
Dr_pglyrp2_R	CTGGGCAGCTGGTGGTTACT	Oehlers et al., 2011
Dr_pglyrp5_F	GACACACAAACACCGTGGACAT	Oehlers et al., 2011
Dr_pglyrp5_R	CCCCATCCTCTGCCTTCATA	Oehlers et al., 2011
Dr_cpa4_F	GGATTCAGGGCTGGATTTCTG	
Dr_cpa4_R	CTCATCCAAAAGGTCCTGCAC	
Dr_cpb1_F	GTCAAGGTGATGATTGATAATCTTCAG	
Dr_cpb1_R	GTATTCTTTCCAATCTTCAGAAGGTGC	
Dr_cel.2_F	GATGCACCTGGTAACTATGGAC	
Dr_cel.2_R	GGTGAAATAATCTGGAAGTTGACACTG	
Dr_tnfaip3_F	GAACCAACGGAGATGGGAATTG	
Dr_tnfaip3_R	CTCCCAGTTCAGCGTGCTG	
Dr_18S_F	CACTTGTCCCTCTAAGAAGTTGCA	Kanther et al., Gastro., 2011
Dr_18S_R	GGTTGATTCCGATAACGAACGA	Kanther et al., Gastro., 2011
Dr_mpx_1F	TCCAAAGCTATGTGGGATGTGA	Kanther et al., Gastro., 2011
Dr_mpx_1R	GTCGTCCGGCAAAACTGAA	Kanther et al., Gastro., 2011
Dr_ncf2_F1	CTGGATGCCATTCTGAAACATAAGCTG	Kanther et al., 2014
Dr_ncf2_R1	GATGGAACATTGTCTATTTGAGGCTG	Kanther et al., 2014
Dr_il1b_F	TGGACTTCGCAGCACAAAATG	Kanther et al., 2014
Dr_il1b_R	GTTCACTTCACGCTCTTGGATG	Kanther et al., 2014
Dr_tnfa_F	GCGCTTTTCTGAATCCTACG	Marjoram et al., 2015
Dr_tnfa_R	TGCCCAGTCTGTCTCCTTCT	Marjoram et al., 2015
Dr_saa_F	CGCAGAGGCAATTCAGAT	Kanther et al., Gastro., 2011
Dr_saa_R	CAGGCCTTTAAGTCTGTATTTGTTG	Kanther et al., Gastro., 2011
Dr_nfkbiab_F	CTCACCGAGGACGGAGACA	Kanther et al., Gastro., 2011
Dr_nfkbiab_R	CTCTTCGGGATAACGCAATCA	Kanther et al., Gastro., 2011
Dr_p53_F1	ATAAGAGTGGAGGGCAATCAGCGA	
Dr_p53_R1	AGTGATGATTGTGAGGATGGGCCT	
Dr_baxa_F1	CGGAGATGAGCTGGATGGAAA	
Dr_baxa_r1	GAAAAGCGCCACAACTCTTCC	
Dr_bcl2a_F1	TCTTCGAGTTTGGTGGGACCATGT	Ji W., et al, J. App. Tox, 2012
Dr_bcl2a_R1	TACATCTCCACGAAGGCATCCCAA	Ji W., et al, J. App. Tox, 2012
Dr_fabp2_F	TCAACGGGACCTGGAAAGTC	Oehlers et al., 2011
Dr_fabp2_R	CCCATTTGTTCCATGAACTTCTC	Oehlers et al., 2011
mCherry_F	CCCCGTAATGCAGAAGAAGA	
mCherry_R	TCTTGGCCTTGTAGGTGGTC	
Dr_cldn15la_F	CACCACATCGACCCTGTATG	Clelland ES, et al.Gen and Comp Endo., 2010
Dr_cldn15la_R	TACCGGCTATTCTGCCTTTG	Clelland ES, et al.Gen and Comp Endo., 2010
