# Supplementary material for: Intestinal Serum amyloid A suppresses systemic neutrophil activation and bactericidal activity in response to microbiota colonization
Source: PLoS Pathog. 2019 Mar 7;15(3):e1007381. doi: 10.1371/journal.ppat.1007381 (PMC6405052; doi:10.1371/journal.ppat.1007381)
Supplement: S2 Table — Bacterial communities in WT and saa-/- mutant (MUT) guts were not significantly different by any of these metrics, whereas WT/MUT guts were significantly different from their housing water (H2O) as expected. (PDF) [file ppat.1007381.s010.pdf]

S2 Table. Statistical analysis of alpha-diversity comparisons from 16S rRNA gene sequencing (Kruskal-Wallis test).

Chao1

Group 1	Group 2	H	p-value	q-value
6d.H2O (n=8)	6d.MUT (n=15)	10.84821958	0.000988908	0.002439429
6d.H2O (n=8)	6d.WT (n=14)	7.835169492	0.005123935	0.006987184
6d.MUT (n=15)	6d.WT (n=14)	0.09340235	0.759895529	0.759895529
70d.H2O (n=5)	70d.MUT (n=12)	10	0.001565402	0.002609004
70d.H2O (n=5)	70d.WT (n=15)	10.73042169	0.001053884	0.002439429
70d.MUT (n=12)	70d.WT (n=15)	0.729835014	0.392936672	0.421003577

Evenness

Group 1	Group 2	H	p-value	q-value
6d.H2O (n=8)	6d.MUT (n=15)	10.41666667	0.001248831	0.006020723
6d.H2O (n=8)	6d.WT (n=14)	7.453416149	0.006331617	0.010552694
6d.MUT (n=15)	6d.WT (n=14)	0.007619048	0.930443263	0.930443263
70d.H2O (n=5)	70d.MUT (n=12)	5.377777778	0.02039484	0.03059226
70d.H2O (n=5)	70d.WT (n=15)	4.954285714	0.026026074	0.035490101
70d.MUT (n=12)	70d.WT (n=15)	2.002380952	0.15705234	0.196315425

Faith Phylogenetic Diversity

Group 1	Group 2	H	p-value	q-value
6d.H2O (n=8)	6d.MUT (n=15)	15	0.000107511	0.000403167
6d.H2O (n=8)	6d.WT (n=14)	13.58385093	0.00022814	0.000570349
6d.MUT (n=15)	6d.WT (n=14)	0.121904762	0.726977734	0.726977734
70d.H2O (n=5)	70d.MUT (n=12)	10	0.001565402	0.002348103
70d.H2O (n=5)	70d.WT (n=15)	10.71428571	0.001063115	0.001989576
70d.MUT (n=12)	70d.WT (n=15)	0.952380952	0.329113986	0.352622128

Observed OTUs

Group 1	Group 2	H	p-value	q-value
6d.H2O (n=8)	6d.MUT (n=15)	10.84821958	0.000988908	0.002439429
6d.H2O (n=8)	6d.WT (n=14)	7.835169492	0.005123935	0.006987184
6d.MUT (n=15)	6d.WT (n=14)	0.09340235	0.759895529	0.759895529
70d.H2O (n=5)	70d.MUT (n=12)	10	0.001565402	0.002609004
70d.H2O (n=5)	70d.WT (n=15)	10.73042169	0.001053884	0.002439429
70d.MUT (n=12)	70d.WT (n=15)	0.729835014	0.392936672	0.421003577

Shannon

Group 1	Group 2	H	p-value	q-value
6d.H2O (n=8)	6d.MUT (n=15)	5.4	0.020136752	0.037756409
6d.H2O (n=8)	6d.WT (n=14)	4.770186335	0.028956691	0.048261152
6d.MUT (n=15)	6d.WT (n=14)	0.007619048	0.930443263	0.983453831
70d.H2O (n=5)	70d.MUT (n=12)	8.711111111	0.003162764	0.012805467
70d.H2O (n=5)	70d.WT (n=15)	6.63047619	0.010024846	0.025062115
70d.MUT (n=12)	70d.WT (n=15)	1.60952381	0.204558753	0.278943754

Simpson

Group 1	Group 2	H	p-value	q-value
6d.H2O (n=8)	6d.MUT (n=15)	5.4	0.020136752	0.050341879
6d.H2O (n=8)	6d.WT (n=14)	6.377329193	0.011558724	0.034676173
6d.MUT (n=15)	6d.WT (n=14)	0.007619048	0.930443263	0.930443263
70d.H2O (n=5)	70d.MUT (n=12)	7.511111111	0.006131953	0.022994825
70d.H2O (n=5)	70d.WT (n=15)	7.56	0.005967799	0.022994825
70d.MUT (n=12)	70d.WT (n=15)	1.866666667	0.171857339	0.214821674
