# Supplementary material for: Intestinal Serum amyloid A suppresses systemic neutrophil activation and bactericidal activity in response to microbiota colonization
Source: PLoS Pathog. 2019 Mar 7;15(3):e1007381. doi: 10.1371/journal.ppat.1007381 (PMC6405052; doi:10.1371/journal.ppat.1007381)
Supplement: S3 Table — Bacterial communities in WT and saa-/- mutant (MUT) guts were not significantly different by any of these metrics, whereas WT/MUT guts were significantly different from their housing water (H2O) as expected. (PDF) [file ppat.1007381.s011.pdf]

S3 Table: Statistical analysis of beta-diversity comparisons from 16S rRNA gene sequencing (PERMANOVA).

Unweighted UNIFRAC

Group 1	Group 2	Sample size	pseudo-F	p-value	q-value
6d.H2O	6d.MUT	23	9.710676626	0.001	0.001363636
6d.H2O	6d.WT	22	9.665690878	0.001	0.001363636
6d.MUT	6d.WT	29	0.916930734	0.553	0.553
70d.H2O	70d.MUT	17	10.81391064	0.002	0.002307692
70d.H2O	70d.WT	20	12.37290625	0.001	0.001363636
70d.MUT	70d.WT	27	1.275344245	0.119	0.1275

Weighted UNIFRAC

Group 1	Group 2	Sample size	pseudo-F	p-value	q-value
6d.H2O	6d.MUT	23	11.72303376	0.001	0.0015
6d.H2O	6d.WT	22	10.35076436	0.001	0.0015
6d.MUT	6d.WT	29	0.393271902	0.934	0.934
70d.H2O	70d.MUT	17	8.998130913	0.001	0.0015
70d.H2O	70d.WT	20	10.93560994	0.001	0.0015
70d.MUT	70d.WT	27	0.396353439	0.816	0.874285714

Bray-Curtis

Group 1	Group 2	Sample size	pseudo-F	p-value	q-value
6d.H2O	6d.MUT	23	8.191011555	0.001	0.001153846
6d.H2O	6d.WT	22	8.314089531	0.001	0.001153846
6d.MUT	6d.WT	29	0.792868311	0.819	0.819
70d.H2O	70d.MUT	17	6.29605003	0.001	0.001153846
70d.H2O	70d.WT	20	7.071145881	0.001	0.001153846
70d.MUT	70d.WT	27	0.449691029	0.801	0.819

Jaccard

Group 1	Group 2	Sample size	pseudo-F	p-value	q-value
6d.H2O	6d.MUT	23	5.264493744	0.001	0.00125
6d.H2O	6d.WT	22	5.400166784	0.001	0.00125
6d.MUT	6d.WT	29	0.916218743	0.661	0.661
70d.H2O	70d.MUT	17	7.533691	0.001	0.00125
70d.H2O	70d.WT	20	7.692192669	0.001	0.00125
70d.MUT	70d.WT	27	1.174809096	0.217	0.2325
